# BAFF Receptor Deficiency Limits Gammaherpesvirus Infection

**DOI:** 10.1128/JVI.03497-13

**Published:** 2014-04

**Authors:** Bruno Frederico, Janet S. May, Stacey Efstathiou, Philip G. Stevenson

**Affiliations:** a Division of Virology, Department of Pathology, University of Cambridge, Cambridge, United Kingdom; b SASVRC/CMVC, University of Queensland, Royal Children's Hospital, Herston, Brisbane, Queensland, Australia

## Abstract

Lymphocyte colonization by gammaherpesviruses (γHVs) is an important target for cancer prevention. However, how it works is not clear. Epstein-Barr virus drives autonomous B cell proliferation *in vitro* but *in vivo* may more subtly exploit the proliferative pathways provided by lymphoid germinal centers (GCs). Murid herpesvirus 4 (MuHV-4), which realistically infects inbred mice, provides a useful tool with which to understand further how a γHV colonizes B cells *in vivo*. Not all γHVs necessarily behave the same, but common events can with MuHV-4 be assigned an importance for host colonization and so a potential as therapeutic targets. MuHV-4-driven B cell proliferation depends quantitatively on CD4^+^ T cell help. Here we show that it also depends on T cell-independent survival signals provided by the B cell-activating factor (BAFF) receptor (BAFF-R). B cells could be infected in BAFF-R^−/−^ mice, but virus loads remained low. This corresponded to a BAFF-R-dependent defect in GC colonization. The close parallels between normal, antigen-driven B cell responses and virus-infected B cell proliferation argue that *in vivo*, γHVs mostly induce infected B cells into normal GC reactions rather than generating large numbers of autonomously proliferating blasts.

**IMPORTANCE** γHVs cause cancers by driving the proliferation of infected cells. B cells are a particular target. Thus, we need to know how virus-driven B cell proliferation works. Controversy exists as to whether viral genes drive it directly or less directly orchestrate the engagement of normal, host-driven pathways. Here we show that the B cell proliferation driven by a murid γHV requires BAFF-R. This supports the idea that γHVs exploit host proliferation pathways and suggests that interfering with BAFF-R could more generally reduce γHV-associated B cell proliferation.

## INTRODUCTION

Gammaherpesviruses (γHVs) persist in lymphocytes and cause lymphocytic cancers. Therefore, it is important to understand how their lymphocyte infections work. The standard model for many years has been *in vitro* B cell transformation by Epstein-Barr virus (EBV) (1), which is independent of normal lymphoid tissue organization. However, *in vivo* EBV persists not in proliferating blasts but in resting memory B cells (2, 3) that show evidence of passage through germinal centers (GCs) (4). Thus, there is discrepancy between *in vitro* and *in vivo* virus behaviors. *In vitro* infections place viral genes outside the context in which they have evolved to function, but at the same time, human analyses are limited in their sampling and capacity to establish cause and effect. Therefore, resolving the discrepancy is not straightforward.

Related γHVs provide another source of information. Those that infect experimentally tractable mammals are particularly useful for establishing cause and effect in a realistic context. Murid herpesvirus 4 (MuHV-4) is a well-characterized example. Despite immortalizing only fetal B cells *in vitro* (5), it colonizes adult lymphoid GCs *in vivo* (6) to establish a persistent infection of memory B cells (7–9). The Kaposi's sarcoma-associated herpesvirus (KSHV) also colonizes B cells *in vivo* (10) and fails to transform them *in vitro*. Thus, EBV, KSHV, and MuHV-4 differ *in vitro* but remain strikingly similar in host colonization. MuHV-4 therefore provides an opportunity to understand functionally in inbred laboratory mice how many γHVs may interact with B cells *in vivo* (11–13). There is no guarantee that every γHV acts in the same way, but with MuHV-4 we can establish a relatively complete functional framework onto which the more fragmented information about human infections can be mapped.

MuHV-4 drives B cell activation and proliferation greatly in excess of antigen-specific responses (14, 15). However, both depend on CD4^+^ T cells (16), CD40 ligand (17), and CD40 (18), implying a similar need for T cell-derived survival signals. Antigen-specific responses also require T cell-independent survival signals, of which those delivered by B cell-activating factor (BAFF) through its main receptor (BAFF-R) have central importance (19, 20). The BAFF-R-deficient phenotype was defined first in AsWyn/J mice (21), in which C-terminal receptor disruption creates a dominant negative mutant (22): transitional B cells developing in the bone marrow fail to survive or undergo T1 to T2 maturation. BAFF-R is also required for follicular B cell survival. Thus, competition for limiting amounts of BAFF regulates circulating B cell numbers. B1 B cells are preserved without BAFF-R, but B2 numbers are severely reduced and marginal-zone B cells are essentially absent (23). IgM responses are still made, but GCs form only transiently and IgG responses are weak (24, 25). Targeted BAFF-R (26) and BAFF knockouts show similar phenotypes (20).

BAFF-R signaling works in part through the induction of antiapoptotic *bcl-2* family members (27). γHVs encode *bcl-2* homologs and inhibit mitochondrial apoptosis pathways (28), so infected B cells might be expected to show independence of BAFF-R-mediated homeostatic control; conversely, extensive reliance on normal B cell physiology (29) would keep virus-driven lymphoproliferation BAFF-R dependent. Therefore, to understand better how γHV host colonization works, we determined the extent to which it depends on BAFF-R.

## MATERIALS AND METHODS

### Mice.

C57BL/6J (Harlan U.K.) and BAFF-R^−/−^ mice (26) (kindly provided by Andrew Sage and Lauren Baker, Division of Cardiovascular Medicine, Cambridge University Medical School) were maintained at the Cambridge University Department of Pathology animal unit and infected with MuHV-4 when 6 to 12 weeks old, either intranasally (i.n.) in 30 μl of Dulbecco's modified Eagle's medium (DMEM) under isoflurane anesthesia (10^4^ PFU) or intraperitoneally (i.p.) in 100 μ1 of DMEM (10^5^ PFU). All animal experiments were approved by the Cambridge University Ethical Review Board and by the 1986 Animal Scientific Procedures Act (project license 80/2538).

### Cells and viruses.

BHK-21 cells (American Type Culture Collection CCL-10) and 3T3-ORF50 cells (30) were grown in Dulbecco's modified Eagle's medium, 2 mM glutamine, 100 U/ml penicillin, 100 mg/ml streptomycin, and 10% fetal calf serum (PAA Laboratories). Wild-type (WT) and EF1α-eGFP MuHV-4 (31) were grown on BHK-21 cells, and their titers were determined. ORF50-deficient MuHV-4 was grown on and its titer determined on 3T3-ORF50 cells (30). Virions were harvested from infected cell supernatants by ultracentrifugation (35,000 × *g*; 90 min), and cell debris was removed by low-speed centrifugation (500 × *g*; 10 min).

### Infectivity assays.

Infectious virus was measured by plaque assay. Virus stocks or organ homogenates were incubated with BHK-21 cells (2 h; 37°C) and then overlaid with 0.3% carboxymethylcellulose. Four days later, the cells were fixed (4% formaldehyde) and stained (0.1% toluidine blue) for plaque counting. Latent plus infectious virus was measured by infectious center assay: single-cell suspensions of explanted spleens, lymph nodes (LNs), and peritoneal washes were obtained postmortem and cocultured with BHK-21 cell monolayers that were then fixed and stained after 4 days. Typically, <5% of the total virus recoverable from lymphoid tissue or peritoneal washes is detected by plaque assay, so the infectious center assay measures mainly latency. Statistical comparisons were by Student's 2-tailed unpaired *t* test unless stated otherwise.

### Viral genome quantitation.

MuHV-4 genomic coordinates 4166 to 4252 were amplified by PCR from 50 to 80 ng DNA of organ homogenates (Rotor-Gene 3000; Corbett Research). PCR products were quantitated by hybridization with a TaqMan probe (genomic coordinates 4218 to 4189) and converted to genome copies by comparison with a standard curve of cloned plasmid template amplified in parallel. Cellular DNA was quantitated in the same reaction by amplifying part of the adenosine phosphoribosyl transferase (APRT) gene, again with TaqMan probe hybridization and template dilutions amplified in parallel. Viral DNA loads were then normalized by the cellular genome copy number of each sample (32).

### Immunohistochemistry and *in situ* hybridization.

Spleens were fixed in phosphate-buffered saline (PBS)–4% formaldehyde (24 h; 4°C), dehydrated in 70% ethanol, and embedded in paraffin. Seven-micrometer sections were dewaxed in xylene and hydrated in graded ethanol solutions. Endogenous peroxidase activity was quenched in PBS–3% H_2_O_2_ (10 min; 23°C). Sections were then blocked with an avidin/biotin blocking kit (Vector Laboratories) and PBS–2% bovine serum albumin (BSA)–2% rabbit serum (1 h; 23°C). B cells were detected with anti-B220 (RA3-6B2; Abcam), biotinylated goat anti-rabbit IgG polyclonal antibody (pAb) (Vector Laboratories), and the Vectastain Elite ABC peroxidase system. All antibody incubations were for 1 h at room temperature, and the sections were washed 3 times in PBS after each incubation. Detection was with ImmPACT DAB substrate (5 min; 23°C; Vector). Viral tRNA/microRNAs (miRNAs) 1 to 4 were detected by *in situ* hybridization (6). After dewaxing and rehydration, fixed sections were treated with proteinase K (100 μg/ml; 10 min; 37°C), acetylated with 25% acetic anhydride in 0.1 M triethanolamine, and then hybridized in 50% formamide–10 mM Tris, pH 7.5 (58°C; 18 h), with a digoxigenin-labeled riboprobe complementary to the tRNA/miRNA transcripts generated by T7 transcription of pEH1.4. The hybridized probe was detected with alkaline phosphatase-conjugated antidigoxigenin Fab fragments (Boehringer Ingelheim) and BCIP–NBT (5-bromo-4-chloro-3-indolylphosphate–Nitro Blue Tetrazolium) substrate. Sections were then counterstained with Mayer's hemalum, dehydrated in ethanol, and mounted in DPX (BDH).

### Immunofluorescence.

Organs were fixed in 1% formaldehyde–10 mM sodium periodate–75 mM l-lysine (24 h; 4°C), equilibrated in 30% sucrose (18 h; 4°C), and then frozen in optimal cutting temperature (OCT) matrix compound. Nine-micrometer sections were cut and then air dried (1 h; 23°C), blocked with 0.3% Triton X-100–5% normal goat serum (1 h; 23°C), and incubated (18 h; 4°C) with antibodies to enhanced green fluorescent protein (eGFP) (rabbit pAb; Abcam), macrophage receptor with collagenous structure (MARCO) (ED31; Serotec), IgM (biotin-conjugated goat pAb; Southern Biotech), IgD (11-26c; Southern Biotech), B220 (RA3-6B2; Abcam), and CD169 (3D6.112; Serotec).

GC B cell staining was with fluorescein-conjugated peanut agglutinin (PNA) (BD Biosciences). Sections were washed 3 times in PBS, incubated (1 h; 23°C) with Alexa 633-conjugated goat anti-rat IgG pAb, streptavidin-conjugated Alexa 568, and Alexa 488- or 568-conjugated goat anti-rabbit IgG pAb (Invitrogen), washed 3 times in PBS, and mounted in Prolong Gold plus DAPI (4′,6-diamidino-2-phenylindole) (Invitrogen). Fluorescence was visualized with a Leica TCS SP5 confocal microscope and analyzed with ImageJ.

## RESULTS

### Intranasal infection.

**FIG 1:**
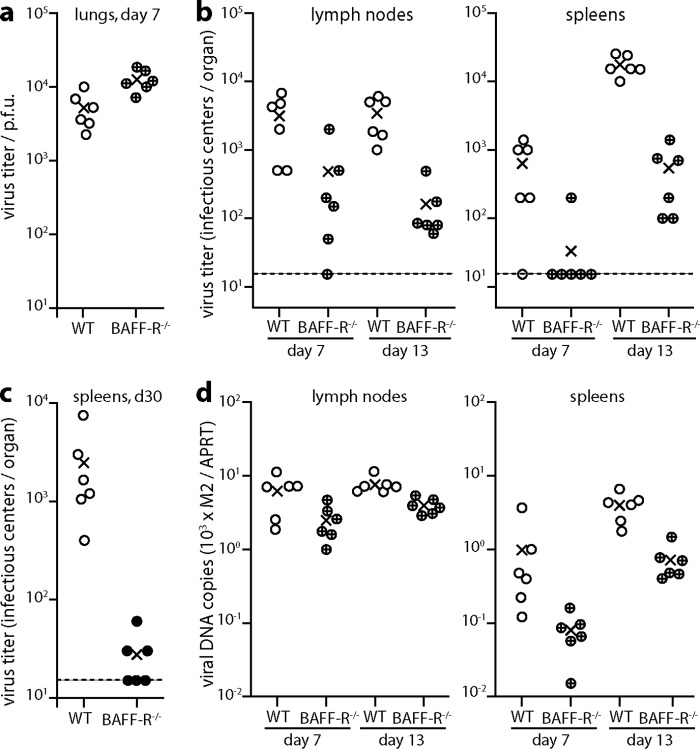
Impaired colonization of BAFF-R^−/−^ mice by i.n. MuHV-4. (a) C57BL/6J (WT) or BAFF-R^−/−^ mice were infected i.n. with MuHV-4 (10^4^ PFU). Seven days later, titers of infectious virus in lungs were detemined by plaque assay. Circles show individual mice; crosses show means. BAFF-R^−/−^ lung titers were significantly higher than those of WT mice (*P* < 0.01). (b) Mice were infected as for panel a, and virus loads in mediastinal lymph nodes and spleens were determined 7 and 13 days later by infectious center assay. Circles show individual mice; crosses show means. The dashed lines indicate the lower limit of assay sensitivity. At day 13, WT titers were significantly higher than those of BAFF-R^−/−^ mice in both lymph nodes (*P* < 0.001) and spleens (*P* < 0.0001). (c) Mice were infected as for panel a, and virus loads in spleens were determined 30 days later by infectious center assay. Circles show individual mice; crosses show means. The dashed line indicates the lower limit of assay sensitivity. WT titers remained significantly higher than those of BAFF-R^−/−^ mice (*P* < 0.05). (d) The organs of mice for panel b were assayed for viral DNA by Q-PCR. Viral genome loads (M2) are normalized by the cellular genome load of each sample (APRT). At day 13, viral DNA loads were significantly higher in WT spleens and lymph nodes than in those of BAFF-R^−/−^ mice (*P* < 0.003).

We infected BAFF-R^−/−^ or wild-type (WT) mice intranasally (i.n.) with MuHV-4 and determined virus titers at day 7, the peak of lytic infection, and at day 13, the peak of latent infection (Fig. 1a). The day 7 lytic virus titers in BAFF-R^−/−^ mouse lungs were significantly higher than those in lungs of WT mice, possibly due to the immunodeficiency of BAFF-R^−/−^ mice. By contrast, latent viral titers in BAFF-R^−/−^ lymph nodes (LNs) and spleens were reduced 10- to 100-fold. BAFF-R^−/−^ lymphoid infections were not completely ablated, and spleen titers increased from day 7 to day 13, but the absolute amounts of infection remained low. Viral DNA loads were also significantly lower in BAFF-R^−/−^ lymphoid tissue than in that of WT mice (Fig. 1b), implying that the lower infectious center assay titers reflected fewer infected cells rather than impaired virus reactivation. After 30 days (Fig. 1c), BAFF-R^−/−^ spleen colonization remained significantly below that of WT mice. Thus, there was a long-term defect in lymphoid infection after i.n. infection rather than just a delay.

### Intraperitoneal infection.

**FIG 2:**
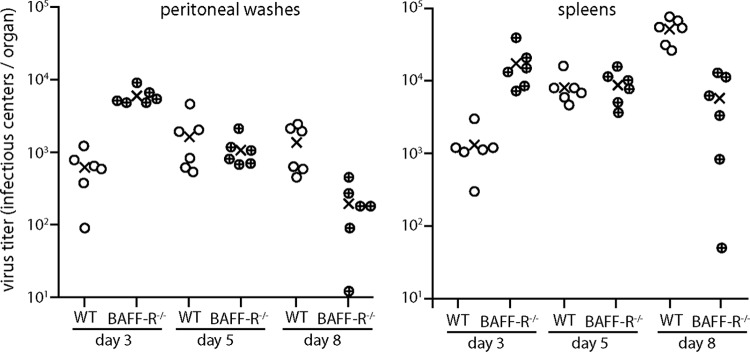
Impaired colonization of BAFF-R^−/−^ mice by i.p. MuHV-4. C57BL/6J (WT) or BAFF-R^−/−^ mice were infected i.p. with MuHV-4 (10^5^ PFU). Virus in peritoneal washes and spleens was then titered by infectious center assay. Circles show individual mice; crosses show means. At day 3, BAFF-R^−/−^ titers were significantly higher in both peritoneal washes (*P* < 0.0001) and spleens (*P* < 0.01). At day 8, WT titers were signifiantly higher in both spleens (*P* < 0.001) and peritoneal washes (*P* < 0.02).

MuHV-4 infection causes marked splenomegaly, and this is where lymphoid infection has been studied in most detail. However, splenic infection by i.n. virus depends on it first reaching LNs (31). Therefore, to determine how much of the BAFF-R^−/−^ spleen infection defect shown in Fig. 1 was specific to this site rather than a consequence of impaired LN infection, we tested splenic colonization more directly by intraperitoneal (i.p.) virus inoculation (Fig. 2). Early on (day 3), BAFF-R^−/−^ mice showed higher virus titers than WT, again possibly due to their immunodeficiency. However BAFF-R^−/−^ titers declined with time, whereas WT titers increased, and by day 8 WT titers were significantly higher. Therefore, despite substantial early virus seeding, spleen infection was poorly amplified in the absence of BAFF-R.

**FIG 3:**
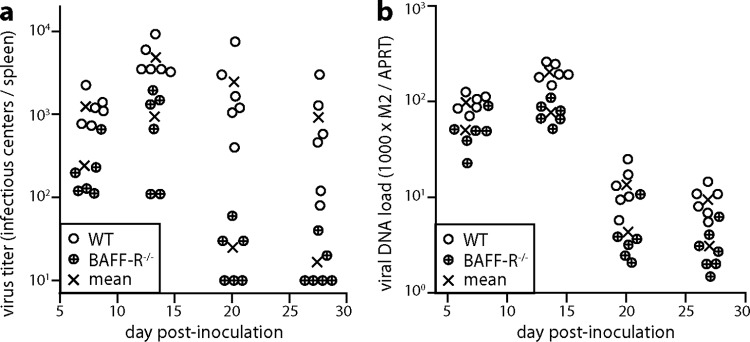
MuHV-4 colonization of i.p.-inoculated BAFF-R^−/−^ mice does not recover with time. (a) We infected C57BL/6J (WT) or BAFF-R^−/−^ mice i.p. with MuHV-4 (10^5^ PFU) and determined the titers of recoverable virus in peritoneal washes and spleens by infectious center assay 7, 13, 20, and 27 days later. Circles show individual mice; crosses show means. BAFF-R^−/−^ titers were significantly lower at all time points (*P* < 0.05). (b) The same samples as for panel a were assayed for viral genome load (M2) relative to cellular genome load (APRT) by Q-PCR. Normalized viral genome loads were significantly lower in BAFF-R^−/−^ mice at all time points (*P* < 0.03).

Infectious center assays over the next 3 weeks (Fig. 3a) showed that BAFF-R^−/−^ spleen colonization remained significantly below that of WT mice. Thus, again there was a fundamental, BAFF-R-dependent infection defect that did not correct with time. The same applied to splenic virus genome loads, measured by quantitative PCR (Q-PCR) (Fig. 3b). BAFF-R^−/−^ mice showed a greater reduction in infectious centers than in viral genome loads. No difference in plaque size was observed, as might be expected for a reactivation defect. Rather, the antibody deficiency of BAFF-R^−/−^ mice may have slowed their clearance of infected cell debris more than that of infectivity, which is achieved mainly by T cells.

### Defective formation of infected B cell follicles in BAFF-R^−/−^ mice.

**FIG 4:**
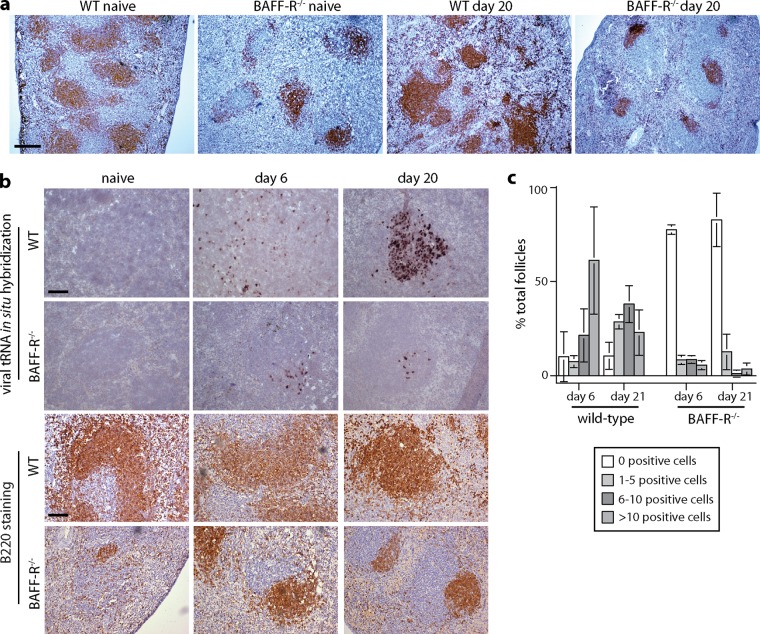
Defective GC formation in MuHV-4-infected BAFF-R^−/−^ mice. (a) Spleen sections from C57BL/6J (WT) and BAFF-R^−/−^ mice were stained for B220 (brown) either before (naive) or 20 days after i.p. MuHV-4 (10^5^ PFU) and counterstained with Mayer's hemalum. The scale bar shows 500 μm. The number of B220 follicles per section was significantly lower in BAFF-R^−/−^ (mean ± standard deviation [SD], 4.7 ± 1.4; *n* = 6) than in WT (14.2 ± 1.5) spleens, both before and after infection (*P* < 10^−6^). (b) We infected C57BL/6J (WT) and BAFF-R^−/−^ mice i.p. with MuHV-4 (10^5^ PFU) and then detected viral tRNA/miRNA transcripts by *in situ* hybridization (dark staining). Examples of positive follicles are shown. B220 staining of the same spleens (brown) relates the extent of tRNA/miRNA staining to the extent of the white pulp occupied by B cells. Scale bars show 100 μm. (c) tRNA/miRNA^+^ cells were counted for spleen sections from 3 mice per group, and follicles were scored as uninfected or infected at a low (1 to 5 cells per section), medium (5 to 10 cells per section), or high (>10 cells per section) level. Bars show means ± SD, counting 20 to 30 follicles per mouse. At both day 6 and day 20, WT spleens contained significantly fewer tRNA/miRNA^+^ follicles than BAFF-R^−/−^ spleens (*P* < 0.0001 by 2-tailed Fisher's exact test), and among the positive follicles, the number of tRNA/miRNA^+^ cells was significantly higher in WT spleens (*P* < 0.01 by Student's unpaired 2-tailed *t* test).

Staining BAFF-R^−/−^ spleens for the B cell marker B220 revealed reduced numbers and sizes of B cell follicles compared to those in WT spleens (Fig. 4a), consistent with BAFF-R being required for GC maintenance as well as mature B cell survival (20). This phenotype was maintained after MuHV-4 infection. Therefore, neither viral gene expression nor the immune stimulation associated with infection compensated for the BAFF-R-dependent GC defect.

*In situ* hybridization for viral tRNA expression showed perifollicular colonization of WT spleens at day 6 and more extensive follicular colonization at day 20 (Fig. 4b). BAFF-R^−/−^ spleens also showed perifollicular viral tRNA expression at day 6. However, there was much less subsequent colonization of follicles, commensurate with the reduced B220 staining. Quantitation across multiple sections (Fig. 4c) established that BAFF-R^−/−^ spleens contained both a significantly higher number of negative follicles and, among the positive follicles, a significantly lower mean number of tRNA^+^ cells. This result confirmed that MuHV-4 could not effectively colonize or amplify the transient GCs of BAFF-R^−/−^ mice.

### Infection tracked by viral eGFP expression.

**FIG 5:**
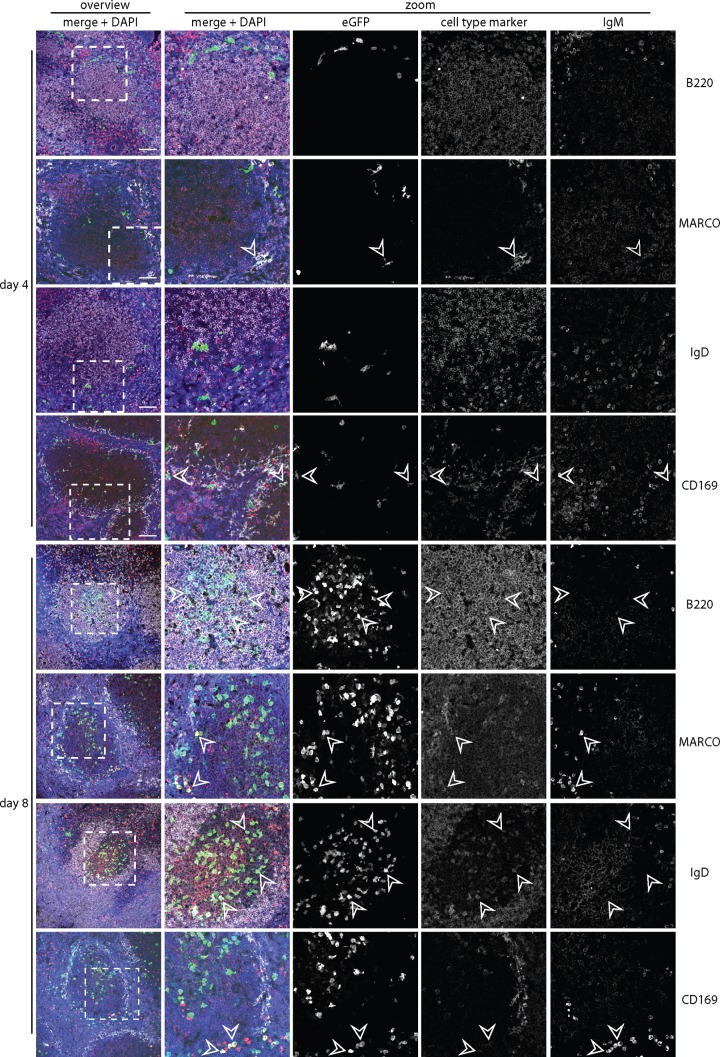
Colonization of C57BL/6J splenic follicles by eGFP^+^ MuHV-4. We infected C57BL/6J mice i.p. with MuHV-4 expressing eGFP from an intergenic EF1α promoter (10^5^ PFU) and 4 or 8 days later identified infection by staining splenic sections for eGFP (green) plus IgM (red; mainly marginal-zone B cells) and, as a further cell type marker, either B220 (mainly follicular B cells), MARCO (marginal-zone macrophages), IgD (mainly follicular B cells), or CD169 (marginal-zone metallophilic macrophages) (white). Nuclei were counter-stained with DAPI (blue). The boxed regions of the left-hand overviews (scale bars show 100 μm) are shown at higher power in the right-hand zoomed images, with either merged or individual channels. Arrows show examples of eGFP^+^ cells.

**FIG 6:**
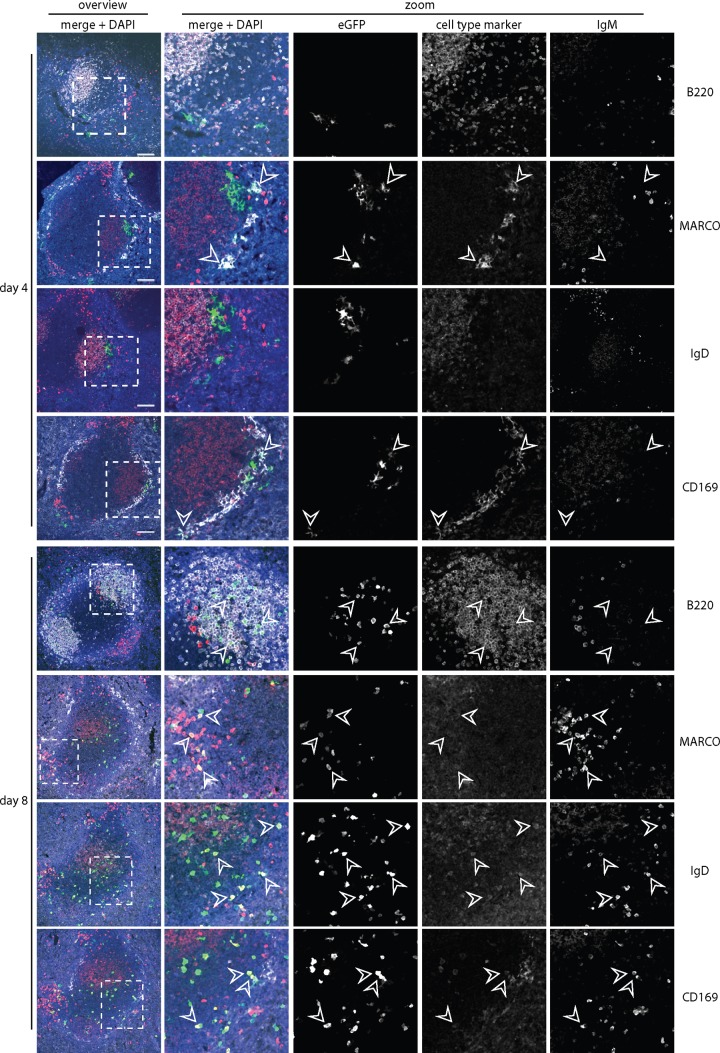
Colonization of BAFF-R^−/−^ splenic follicles by eGFP^+^ MuHV-4. We infected BAFF-R^−/−^ mice i.p. with eGFP^+^ MuHV-4 as for Fig. 5 and 4 or 8 days later stained splenic sections for eGFP (green) plus IgM (red) and, as a further cell type marker, either B220, MARCO, IgD, or CD169 (white). Nuclei were counter-stained with DAPI (blue). The boxed regions of the left-hand overviews (scale bars shows 100 μm) are shown at higher power in the right-hand zoomed images, with either merged or individual channels. Arrows show examples of eGFP^+^ cells.

**FIG 7:**
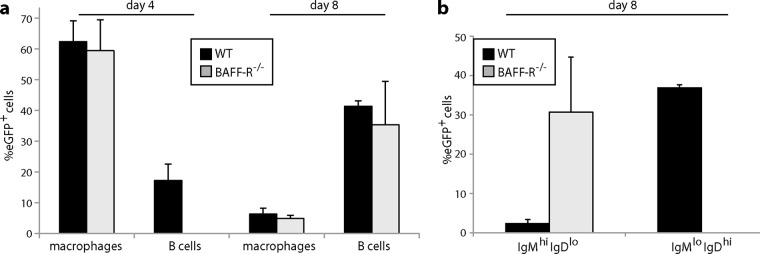
Quantitation of splenic colonization by eGFP^+^ MuHV-4. (a) We infected C57BL/6J (WT) or BAFF-R^−/−^ mice i.p. with eGFP^+^ MuHV-4 as for Fig. 5 and 6 and after 4 or 8 days typed eGFP^+^ cells as macrophages (CD169^+^ or MARCO^+^) or B cells (IgM^+^ or IgD^+^). Each bar shows the mean ± SD for 3 sections from each of 3 mice. Macrophage infection was not significantly different between the mouse groups, but WT mice showed significantly more B cell infection at day 4 (*P* < 0.001). (b) EGFP^+^ B cells at day 8 were further subdivided into IgM^hi^ IgD^lo^ (predominantly marginal-zone) and IgM^lo^ IgD^hi^ (predominantly follicular) populations. The eGFP^+^ B cells of WT mice were significantly higher in IgM^lo^ IgD^hi^ and significantly lower in IgM^hi^ IgD^lo^ than those of BAFF-R^−/−^ mice (*P* < 0.001).

We then tracked spleen infection by lytic cycle-independent eGFP expression from a viral EF1α promoter. Four days after i.p. infection of WT mice (Fig. 5), eGFP was seen mainly in CD169^+^ and MARCO^+^ marginal-zone macrophages; very little was in IgM^+^ or IgD^+^ B cells. After 8 days, relatively few macrophages were eGFP^+^; infection localized instead to B220^+^ B cell follicles, with many eGFP^+^ IgM^+^ and eGFP^+^ IgD^+^ cells. Thus, WT infection started in splenic macrophages and then spread to B cells in GCs. BAFF-R^−/−^ spleens (Fig. 6) also showed marginal-zone macrophage infection after 4 days, and eGFP^+^ Ig^+^ cells were associated with B220^+^ follicles at day 8. However, the follicles were smaller and less numerous than in WT spleens, consistent with the *in situ* hybridization data (Fig. 4b), and the eGFP^+^Ig^+^ cells were more commonly IgM^+^ than IgD^+^ (Fig. 7). Impaired B cell maturation from IgM^hi^ IgD^lo^ (hi indicates high; lo indicates low) to IgM^lo^ IgD^hi^ is well described for BAFF and BAFF-R deficient mice (21, 24). Therefore, infected BAFF-R^−/−^ B cells remained bound by their genetic maturation defect.

**FIG 8:**
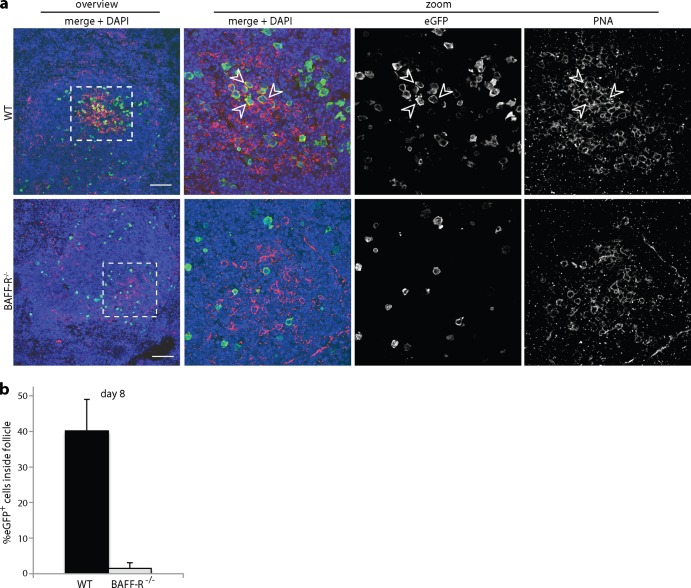
Colonization of GC B cells by eGFP^+^ MuHV-4. (a) We infected C57BL/6J (WT) or BAFF-R^−/−^ mice i.p. with eGFP^+^ MuHV-4 (10^5^ PFU) and 8 days later stained splenic sections for eGFP (green) and PNA (red). Nuclei were stained with DAPI (blue). Arrows show eGFP^+^ PNA^+^ cells. The boxed areas in the left-hand overview images (scale bar = 100 μm) are shown at higher magnification, with separate and merged channels in the right-hand images. No eGFP^+^ PNA^+^ cells were seen in BAFF-R^−/−^ spleens. (b) Mice were infected as for panel a, and eGFP^+^ cells were scored as inside or outside PNA^+^ follicles. Each bar shows the mean ± SD for 3 sections from each of 3 mice. In WT mice, a significantly higher proportion of the eGFP^+^ cells were inside follicles than in BAFF-R^−/−^ mice (*P* < 0.0001).

Peanut agglutinin (PNA) staining at 8 days postinfection (Fig. 8) showed PNA^+^ eGFP^+^ cells in WT but not BAFF-R^−/−^ spleens. The eGFP^+^ WT cells tended to be PNA^int^ (int indicates intermediate) rather than PNA^hi^, and far from all were PNA^+^, but there was a clear difference from the eGFP^+^ BAFF-R^−/−^ cells, which also did not localize to PNA^+^ areas of the spleen (Fig. 8a and b). These data were consistent with MuHV-4 infecting BAFF-R^−/−^ B cells but then failing to overcome their defect in GC formation.

### IgG response to MuHV-4 infection.

**FIG 9:**
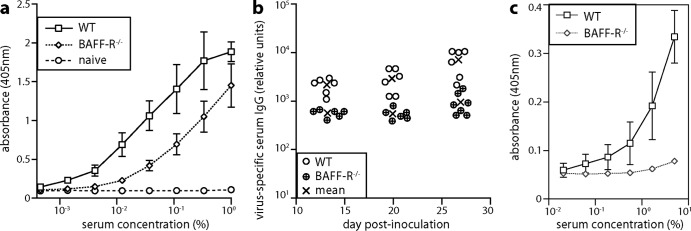
MuHV-4-specific antibody response. (a) We infected C57BL/6J (WT) or BAFF-R^−/−^ mice i.p. with MuHV-4 (10^5^ PFU). Sera taken 27 days later were assayed for MuHV-4-specific IgG by ELISA. Each line shows mean ± SD absorbance readings of sera from 6 mice. naive, uninfected controls (2 mice). (b) Mice were infected as for panel a and bled 13, 20, and 27 days later, and MuHV-4-specific serum IgG was measured by ELISA. Titers were normalized by comparison with a pooled standard immune serum. Circles show individual titers; crosses show means. Although IgG responses were readily detectable in BAFF-R^−/−^ mice, WT titers were significantly higher at each time point (*P* < 0.01). (c) We infected C57BL/6J (WT) or BAFF-R^−/−^ mice i.p. with ORF50-deficient MuHV-4 (10^6^ PFU), which cannot replicate without complementation. Sera taken 14 days later were assayed for MuHV-4-specific IgG by ELISA. Each line shows mean ± SD absorbance readings of sera from 4 mice.

We measured antigen-specific B cell responses in infected WT and BAFF-R^−/−^ mice by enzyme-linked immunosorbent assay (ELISA) for MuHV-4-specific serum IgG (Fig. 9). BAFF-R^−/−^ responses were 10-fold lower than those of WT controls. Thus, BAFF-R^−/−^ mice showed quantitatively similar impairments of antigen-specific B cell responses and virus-driven lymphoproliferation, consistent with these processes having a similar requirement for BAFF-R signaling. To allow for differences in virus load possibly affecting antibody responses, we also tested the response of WT and BAFF-R^−/−^ mice to ORF50-deficient MuHV-4 (Fig. 9c), which fails to replicate without complementation and so elicits virion antigen-specific antibodies only as input virus. Responses were lower than with WT MuHV-4, but again WT mice had a substantially greater response than BAFF-R^−/−^ mice.

## DISCUSSION

EBV analyses have produced two contrasting models of how γHVs behave in B cells: viral genes driving autonomous B cell proliferation (33) and infected B cells hitchhiking along host pathways of proliferation and differentiation (29). EBV certainly can transform B cells, but the *in vitro* cultures in which this is the predominant infection outcome may be unrealistic. MuHV-4 provides functional *in vivo* data to supplement *in vitro* and descriptive *in vivo* analyses of EBV. It drives B cell proliferation poorly in CD4^+^ T cell-deficient (16) and B cell receptor transgenic (34) mice. We showed here that BAFF-R is also important. Thus, host colonization required both T cell and non-T cell host survival signals, consistent with the hitchhiking model. *In vitro* and *in vivo* B cell infections differ in the latter being more exposed to immune attack. Therefore, host-dependent B cell proliferation may predominate *in vivo* because reducing viral gene expression makes lymphoproliferation immunologically less conspicuous.

Infected B cells must still be driven into GCs. They lack obvious antigen specificity (14, 15), so the signal presumably comes from viral genes. The only MuHV-4 genes with clear latency expression profiles are *ORF73* and *M2* (35). *ORF73* encodes the viral episome maintenance protein (EBNA-1 in EBV, LANA in KSHV) (36), while *M2* regulates B cell receptor signaling (like LMP-2A in EBV and K1 in KSHV) (37). The MuHV-4 latency program of ± *ORF73* ± *M2* is therefore analogous to the EBV latency maintenance program of ± EBNA-1 ± LMP-2A (29). This would suggest that as with antigen-driven responses, the stimulus for MuHV-4-infected B cells to enter GCs comes from without.

MuHV-4-driven B cell activation exceeding both antigen-specific responses and B cell infection rates (14, 15) suggests the possibility of superantigen-like B cell activation. This has precedents in murine retroviruses using superantigens to colonize B cells (38), in herpesvirus saimiri producing a T cell superantigen (39), and in EBV inducing a retroviral superantigen (40) and promoting V_H_4-34^+^ B cell expansion (41). Splenic myeloid infection preceding B cell infection implied abundant opportunity for myeloid-derived proteins to promote B cell colonization (42). Infected myeloid cells transcribe *M1*, *M3*, and *M4*, a set of homologous genes for secreted viral glycoproteins (8). M1 drives a superantigen-like Vβ4^+^ CD8^+^ T cell stimulation (43, 44), and both the M3 chemokine binding protein (45) and M4 promote B cell infection (46–48). Thus, lytically infected myeloid cells could drive latently infected B cells into GCs.

Transgenic *bcl-2* expression rescues B cell numbers in BAFF-R-deficient mice (27). MuHV-4—like EBV and KSHV—expresses an antiapoptotic *bcl-2* homolog (*M11*) (49) but failed to overcome the limits on B cell proliferation set by BAFF-R deficiency. MuHV-4 lacking *M11* (*M11*^−^ MuHV-4) has a latency establishment defect (50), but *M11* may not be expressed at a suitable stage of the viral life cycle to substitute for BAFF-R signaling. The myeloid/B cell infection model again provides a possible explanation: *M11* is transcribed in splenic macrophages and dendritic cells rather than follicular or GC B cells (8) and i.p.-inoculated, *M11*-deficient MuHV-4 shows impaired macrophage infection (51). Thus, the latency defect of *M11*^−^ MuHV-4 could be an indirect consequence of impaired myeloid infection rather than a direct consequence of B cell apoptosis. A similar knock-on effect would explain why disrupting the major histocompatibility complex (MHC) class I evasion protein K3—another MuHV-4 lytic gene product (52) that functions in myeloid cells (53)—impairs B cell infection (54). Thus, we hypothesize that myeloid-expressed viral genes foster a suitable microenvironment for host-driven—and therefore BAFF-R-dependent—infected B cell proliferation.

The B1 B cells present in BAFF-R^−/−^ mice could not substitute for B2 B cells in providing a normal reservoir of long-term infection. Nor could the remaining B2 B cells of BAFF-R^−/−^ mice (approximately 10% of WT numbers) proliferate to form a normal reservoir, even though MuHV-4 normally infects only 1 to 2% of mature B cells and any infected B cells managing to traverse transient BAFF-R^−/−^ GCs should then become BAFF-R independent (20). This was consistent with a strong viral reliance on host-driven lymphoproliferation. The implication is that BAFF-R might be a viable target for therapeutic intervention when infection-associated lymphoproliferation causes disease, aiming to reduce lymphoproliferation and the chance of oncogenic host mutations in GCs without the profound immunosuppression of memory B cell depletion.
